# Nitric oxide is involved in hydrogen gas-induced cell cycle activation during adventitious root formation in cucumber

**DOI:** 10.1186/s12870-016-0834-0

**Published:** 2016-06-28

**Authors:** Yongchao Zhu, Weibiao Liao, Lijuan Niu, Meng Wang, Zhanjun Ma

**Affiliations:** College of Horticulture, Gansu Agricultural University, Lanzhou, 730070 People’s Republic of China

**Keywords:** Hydrogen-rich water, Nitric oxide, Cell cycle, Cell cycle-related genes, Adventitious root formation, Cucumber

## Abstract

**Background:**

Adventitious root development is a complex process regulated through a variety of signaling molecules. Hydrogen gas (H_2_) and nitric oxide (NO), two new signaling molecules are both involved in plant development and stress tolerance.

**Results:**

To investigate the mechanism of adventitious root development induced by hydrogen-rich water (HRW), a combination of fluorescence microscopy and molecular approaches was used to study cell cycle activation and cell cycle-related gene expression in cucumber (*Cucumis sativus* ‘Xinchun 4’) explants. The results revealed that the effect of HRW on adventitious root development was dose-dependent, with maximal biological responses at 50 % HRW. HRW treatment increased NO content in a time-dependent fashion. The results also indicated that HRW and NO promoted the G1-to-S transition and up-regulated cell cycle-related genes: *CycA* (A-type cyclin), *CycB* (B-type cyclin), *CDKA* (cyclin-dependent kinase A) and *CDKB* (cyclin-dependent kinase B) expression. Additionally, target genes related to adventitious rooting were up-regulated by HRW and NO in cucumber explants. While, the responses of HRW-induced adventitious root development and increase of NO content were partially blocked by a specific NO scavenger 2-(4-carboxyphenyl)-4,4,5,5-tetramethylimidazoline-1-oxyl-3-oxide potassium salt, NO synthase (NOS)-like enzyme inhibitor N^G^ –nitro-_L_-arginine methylester hydrochloride, or nitrate reductase inhibitors tungstate and NaN_3_. These chemicals also partially reversed the effect of HRW on cell cycle activation and the transcripts of cell cycle regulatory genes and target genes related adventitious root formation.

**Conclusions:**

Together, NO may emerge as a downstream signaling molecule in H_2_-induced adventitious root organogenesis. Additionally, H_2_ mediated cell cycle activation via NO pathway during adventitious root formation.

## Background

Adventitious rooting is a key step in the vegetative propagation of plants. Understanding the mechanism of adventitious rooting is of significant importance to strategize breeding efforts to maximizing its marketable yield [1]. Adventitious root development is a complex process regulated by several lines of environmental and endogenous factors. In recent years, there has been increasing interest in the field of signal transduction during adventitious rooting. Untill now, nitric oxide (NO) [2, 3], Ca^2+^ ions, calmodulin (CaM) [4], Ca^2+^-dependent protein kinase activities (CDPK) [5], cyclic guanosinemonophosphate (cGMP) [6], ethylene [7], mitogen-activated protein kinase [2], carbon monoxide [8], polyamines [9], hydrogen peroxide [10], hydrogen sulfide [11] and hydrogen gas (H_2_) [12] have been suggested to be involved in adventitious rooting process. However, the complex network of signaling molecule associated with adventitious rooting remains unclear. A better understanding of the regulation of initiation of adventitious rooting by signaling molecules will advance our understanding of the molecular mechanisms regulating adventitious root development.

The gaseous compound NO is a redox-active small signaling molecule which may regulate almost all biotic and abiotic stress responses [13, 14]. Previous studies have also demonstrated the involvement of NO in plant various physiological processes such as maturation and senescence [15, 16], seed germination or dormancy [17, 18], floral transition [19] and stomatal movement [20]. NO is also required for root organogenesis, including lateral root formation [21], root hair formation [22], and adventitious rooting [3, 23]. Although numerous studies have demonstrated the involvement of NO in adventitious roots formation, there is currently little information on its mechanism.

H_2_, a colorless, odorless and tasteless gas is the structurally simplest gas in nature and known as an inert gas. Previous studies have demonstrated that H_2_ is a potential therapeutic medical gas [24]. It has received worldwide attention because it could selectively reduce hydroxyl radical and peroxynitrite in cell [25]. Subsequently, accumulated evidence from a variety of animal experiments and clinical tests indicated that H_2_ may act as an anti-inflammatory, anti-apoptotic, and anti-allergic agent [26]. More recently, there have been some reports indicating that H_2_ may play critical roles in plant stress response including salinity [27–29], drought [29, 30], paraquat-induced oxidative stress [30], cadmium toxicity [31], aluminum stress [32], mercury toxicity [33], and UV-A irradiation [34]. Hydrogen-rich water also could delay postharvest ripening and senescence of kiwifruit during storage by regulating the antioxidant defence [35]. Notably, Lin et al. [12] found that H_2_ might regulate cucumber adventitious root development in a heme oxygenase-1/carbon monoxide-dependent manner. The author suggested that exogenous HRW treatment might be a good option to induce plant root organogenesis. However, the mechanism of H_2_ on regulating adventitious root development needs to be fully investigated.

Thus, H_2_ and NO have been considered as signaling modulators with multiple biological functions in plants. Little information is available, however, about the connection between H_2_ and NO in regulating physiological process. It was found that H_2_ inhibited LPS/IFNγ-induced NO production through modulation of signal transduction in macrophages and ameliorates inflammatory arthritis in mice [36]. It provided the molecular basis for H_2_ effects on inflammation and a functional interaction between H_2_ and NO. The interaction effect between H_2_ and NO was also found in alfalfa. Recently, HRW was reported to alleviate aluminum-induced inhibition of root elongation via decreasing NO production [32]. More recently, the crosstalk between H_2_ and NO was reported to play central roles in the ABA signaling cascade during stomatal movement [37].

The cell cycle consists of two major events, cell development which is the critical driving forces in completion of the ontogenic program during plants life cycle. All phases of cell cycle are regulated by the heterodimeric complexes of highly conserved proteins, cyclin-dependent kinase (CDK) and cyclin [38]. In higher eukaryotes, the activation of CYCD and CDKA leads to produce a repressor protein kinase that phosphorylates the retinoblastoma protein at G1-to-S phase transition [39]. In the next checkpoint, B-type CKDs and A-and B-type cyclins are involved in G2-to-M phase transition [40]. In addition, CDK/cyclin complexes are inactivated by a class of CDK-inhibitory proteins [41].

It was suggested that H_2_ and NO have positive effects on adventitious root formation. Our previous results showed that 50 μM NO donor sodium nitroprusside (SNP) had significant effect on adventitious root [10]. However, the crosstalk of NO and H_2_ in promoting adventitious rooting and its mechanism are still puzzled. In the study, molecular and pharmacological approaches were used to investigate the influence of H_2_ and NO on the adventitious root development in cucumber (*Cucumis sativus* ‘Xinchun 4’) explants, as well as cell cycle activation and cell cycle- and adventitious rooting related genes in hypocotyls. Therefore, the objectives of this study are to determine the role of NO in H_2_-regulated cell cycle during adventitious rooting.

## Results

### HRW promoted adventitious root development in a dose-dependent manner

**Fig. 1:**
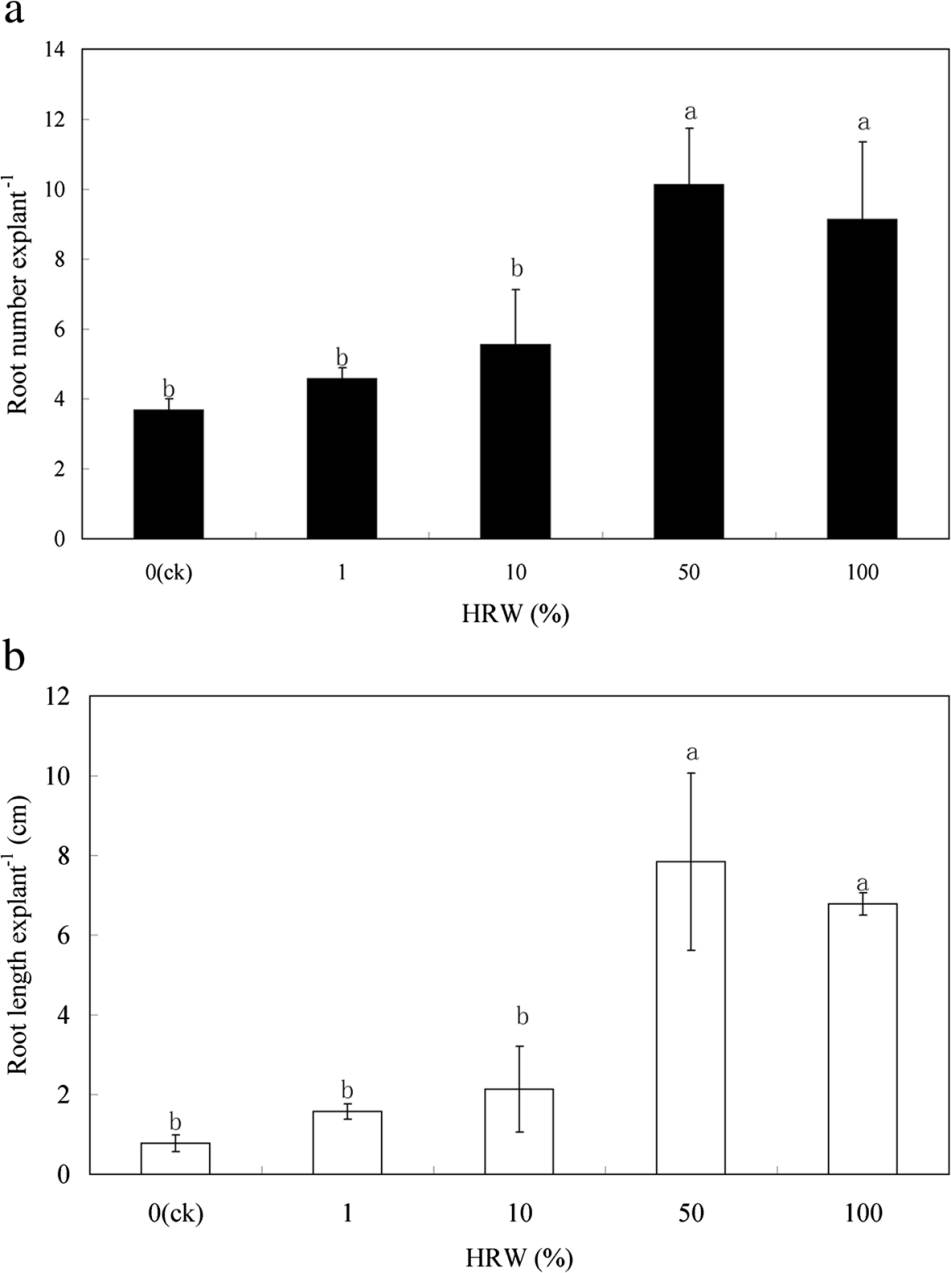
Effect of different concentrations of HRW on the induction of adventitious root development in cucumber explants. The primary root system was removed from hypocotyls of 5-day-old germinated cucumber. Explants were incubated with distilled water or different concentrations of HRWas indicated for 5 days. Adventitious root numbers (**a**) and length (**b**) were expressed as mean ± SE (*n* = 3) 10 explants was used in an independent experiment. *Bars* not sharing the same letters were significantly different by Duncan’s test (*P* < 0.05)

To understand the effect of HRW on adventitious root development, cucumber explants were treated with different concentrations of HRW (0, 1 %, 10 %, 50 %, 100 %). As compared with the control (distilled water), HRW had significant effects on adventitious rooting (Fig. 1). Compared with the control, 1 and 10 % HRW had no significant difference in root number and root length, but the effects were significantly lower than treatments with 50 and 100 % HRW. Among the different concentrations, the maximum root number (10.13) and root length (7.84 cm) were observed with 50 % HRW treatment (Fig. 1). Therefore, the promotions of root development were maximal at 50 % HRW, and this concentration was used further for studies during rooting process.

### The HRW-induced adventitious root development was reversed by NO elimination

**Fig. 2:**
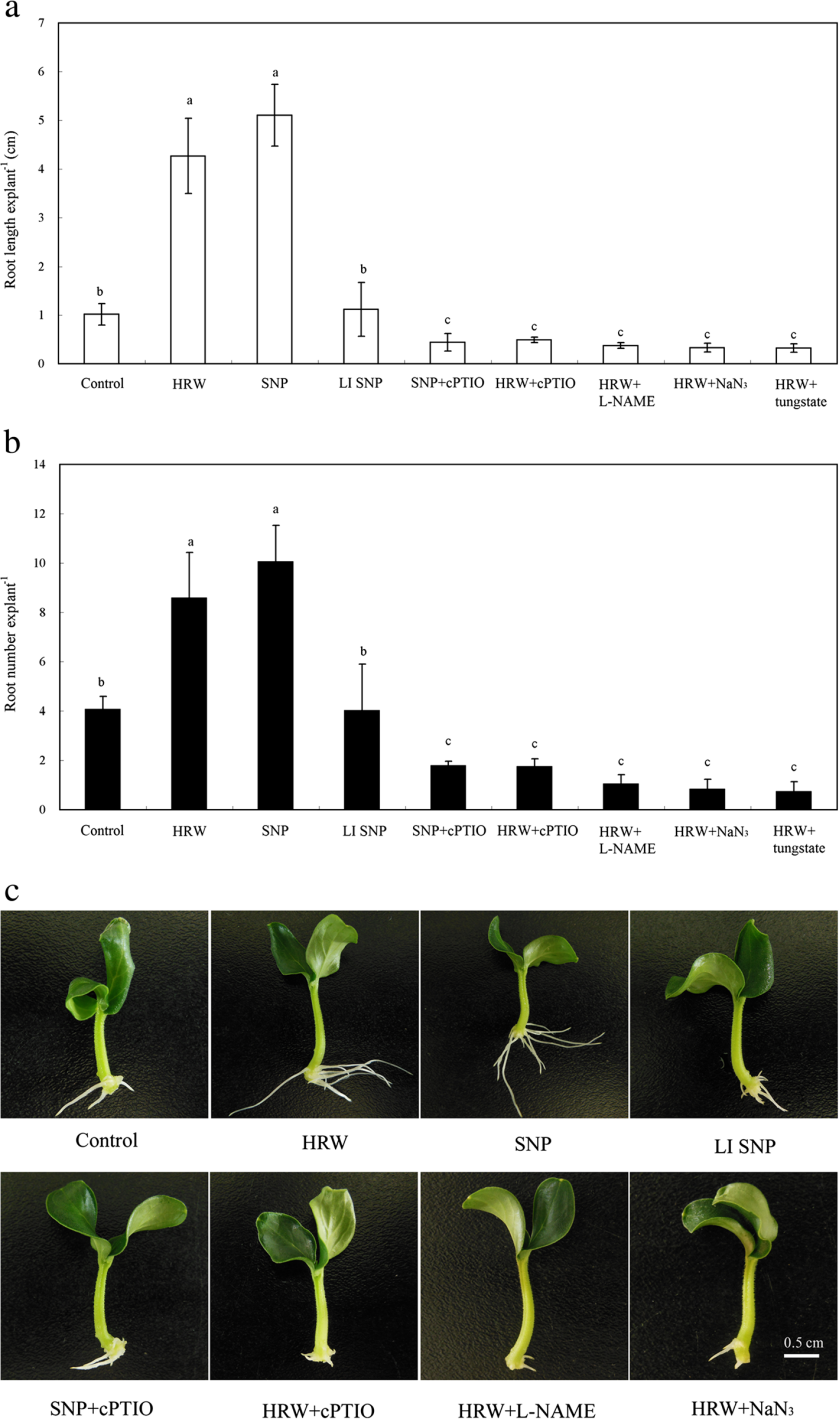
Effect of HRW, SNP, LI SNP, cPTIO, L-NAME and NaN_3_ on adventitious root development in cucumber explants. The primary root system was removed from hypocotyls of 5-day-old germinated cucumber. Explants of cucumber were incubated with 50 % HRW, 50 μM SNP, 50 μM LI SNP, light-inactivated sodium nitroprusside, 200 μM cPTIO, 30 μM L-NAME or 10 μM NaN_3_ as indicated for 5 days. Adventitious root number (**a**) and length (**b**) were expressed as mean ± SE (*n* = 3). 10 explants was used in an independent experiment. Bars not sharing the same letters were significantly different by Duncan’s test (*P* < 0.05). Photographs (**c**) were taken after 5 days of treatment. Bar = 5 cm

SNP induced adventitious root development, however, cPTIO reduced the positive effect of SNP. To further investigate the involvement of NO in H_2_-induced adventitious root development, the effects of NO scavenger cPTIO, NOS inhibitor L-NAME and NR inhibitor NaN_3_ and tungstate on adventitious root development of explants treated with HRW were determined. HRW -induced rooting was partially reversed by cPTIO, L-NAME tungstate or NaN_3_. Compared with the control, explants treated HRW plus with cPTIO, L-NAME or NaN_3_ resulted in a significant decrease root number and length (Fig. 2). Furthermore, other by-products of SNP decomposition had no significant promotion on adventitious root development and it was significantly lower than treatments with the active SNP. The above results demonstrated that NO may act as a downstream signaling molecular in HRW-induced adventitious root development.

### The production of endogenous NO was involved in HRW-induced of adventitious root formation

**Fig. 3:**
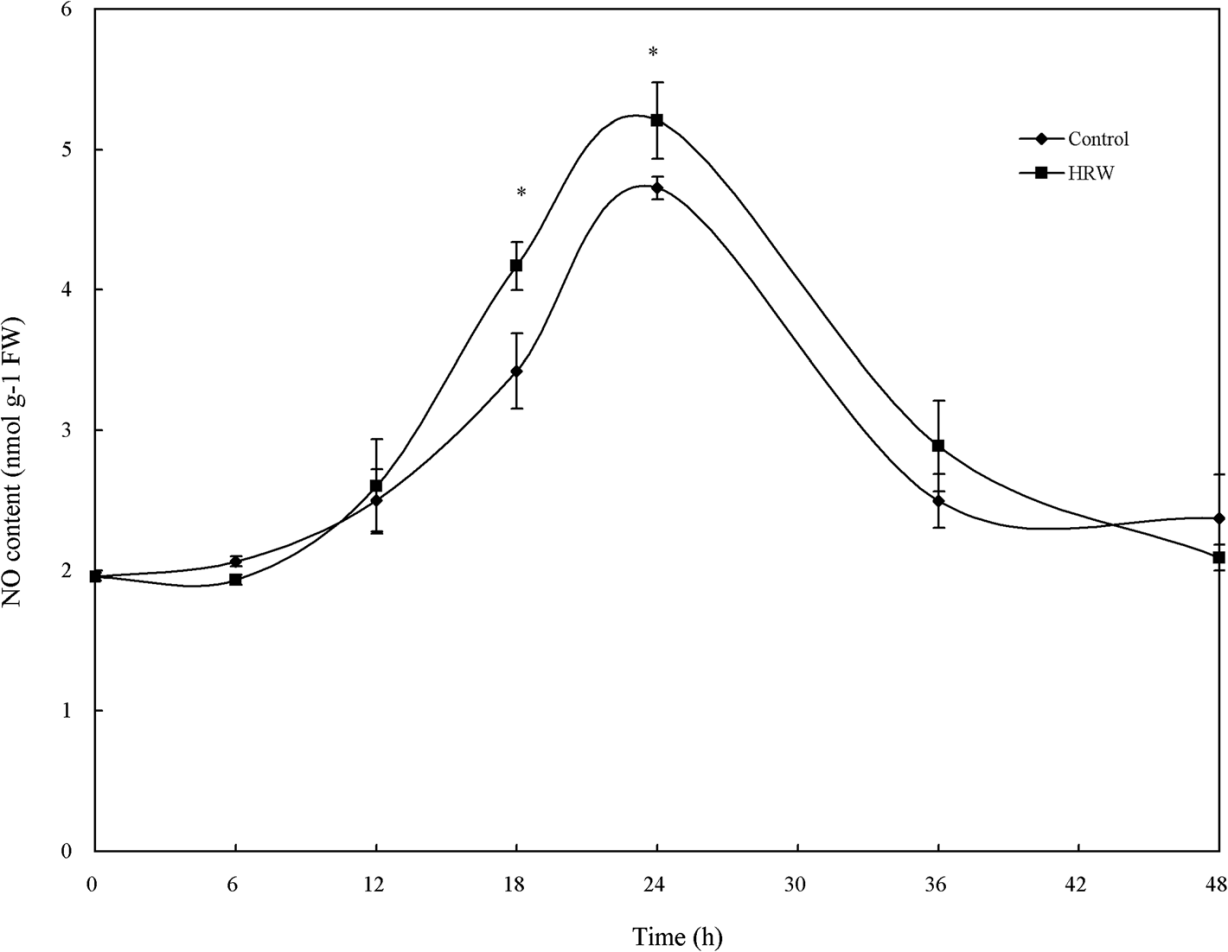
Effect of HRW on the production of endogenous NO during adventitious rooting. The primary root system was removed from the hypocotyls of 5-d-old, germinated cucumber seedlings. NO levels of hypocotyls were determined by Greiss reagent in explants treatment with distilled water (control) or 50 % HRW for 24 h. Values (means ± SE) are the averages of three independent experiments (*n* = 3). Asterisks indicate that mean values are significantly different between the treatments of HRW and Control (*P* < 0.05) according to Duncan’s multiple test

Since NO plays a key role in regulating adventitious root development, we assayed whether H_2_ affects NO production in hypocotyls treated with HRW or with distilled water. As shown in Fig. 3, 50 % HRW induced NO production in a time-dependent manner. After 6 h of HRW treatment, an increase in NO content was observed, reaching a maximum at 24 h of treatment. While, NO content was weakly detected in the control explants, which was significant lower than that in HRW-treated explants at 18 and 24 h (Fig. 3).

### NO may be involved in HRW-induced cell cycle activation during adventitious root development

**Fig. 4:**
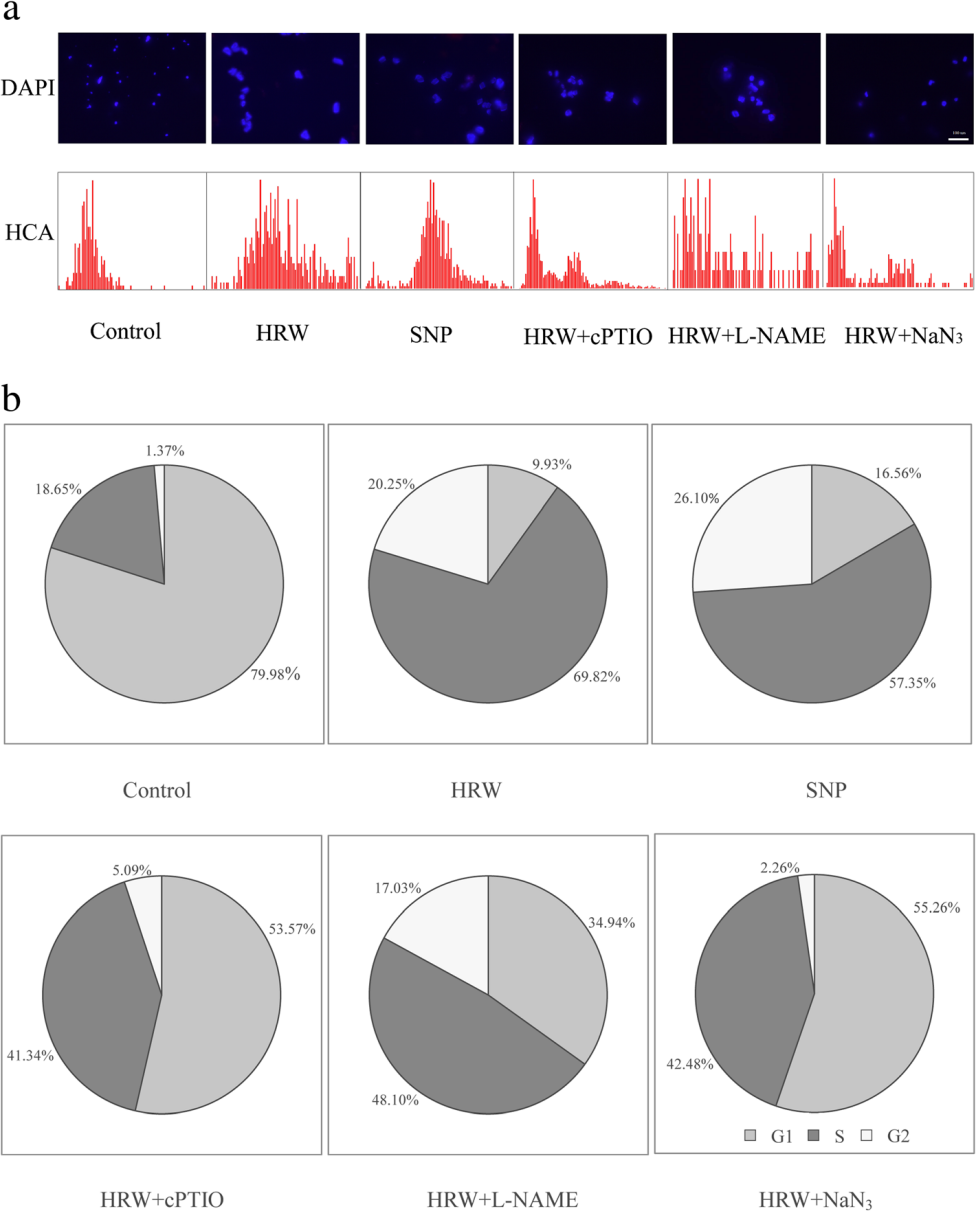
Effect of HRW, SNP, cPTIO, L-NAME and NaN_3_ on cell cycle phase distribution during adventitious rooting. The primary root system was removed from hypocotyls of 5-day-old germinated cucumber. Explants of cucumber were incubated with 50 % HRW, 50 μM SNP, 200 μM cPTIO, 30 μM L-NAME or 10 μM NaN_3_ as indicated for 6 h. Cucumber hypocotyls were fixed by paraformaldehyde and nuclei were extracted by chopping in Galbraith buffer. The nuclei were resulted with DAPI, and then visualized by fluorescent microscopy; the same samples were processed for multiparametric cell cycle high-content analysis (HCA) (**a**). Bar = 100 μm. The relative fluorescence intensity of nuclei was analyzed by Image Pro software (**b**)

To gain insight into the mechanism of HRW-induced adventitious root development, whether NO might be involved in HRW functioning in the induction of cell-division activity was analyzed. Representative pictures of nuclei (DAPI panels), together with DNA histograms obtained from DAPI staining (HCA panels), were shown in Fig. 4a. Compared to the control samples, treatments with 50 % HRW or 50 μM SNP showed much more pronounced foci (Fig. 4a), thereby indicating more cells were at G1-to-S transition phase. However, cPTIO, L-NAME, or NaN_3_ reduced pronounced foci induced by HRW. DNA histograms obtained from DAPI staining, together with representative pictures of nuclei, were shown in Fig. 4a (HCA panels). DNA histograms of asynchronous cells clearly display a S peak in HRW and SNP treatment and a diploid (G1) peak in the control, HRW + cPTIO, HRW + L-NAME and HRW + NaN_3_ treatments.

The highest percentage of cells in G1 phase was found in the control, which were 700.50 % and 634.20 % higher than that in HRW and SNP treatments, respectively (Fig. 4b). However, the percentage of total cell population in HRW and SNP treatments was higher than that in the control (Fig. 4b). Thus, H_2_ and NO promoted G1 to S transition phase, suggesting that a subpopulation of cells in G1 phase was induced to enter a new cell cycle in a synchronous manner. If cPTIO, L-NAME, or NaN_3_ was added to the HRW solution simultaneously, the percentage of cells in G1 phase was increased by 43.64, 25.01 and 45.33 %, but the percentage of cells in S phase was decreased by 28.48, 21.72 and 27.3 % (Fig. 4b).

### NO participates in HRW-mediated transcript levels of cell cycle regulatory genes during adventitious root development

**Fig. 5:**
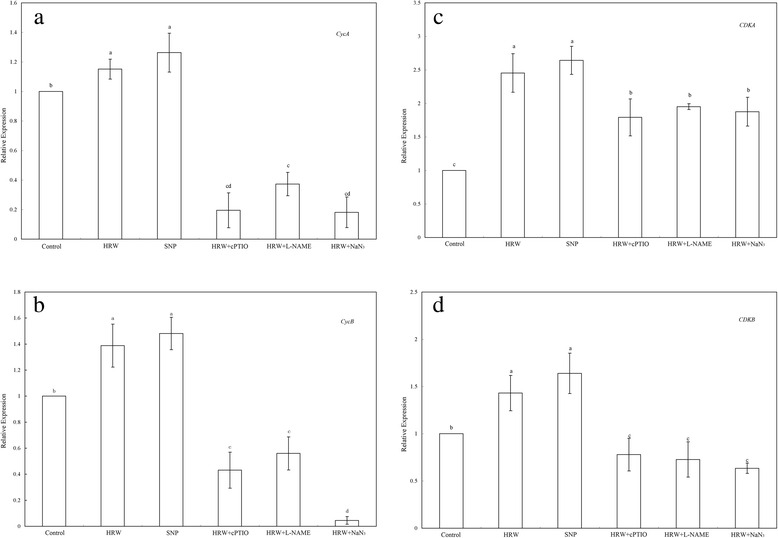
Effect of HRW, SNP, cPTIO, L-NAME and NaN_3_ on the expression of cell cycle-related genes in adventitious rooting. The primary root system was removed from hypocotyls of 5-day-old germinated cucumber. Explants of cucumber were incubated with 50 % HRW, 50 μM SNP, 200 μM cPTIO, 30 μM L-NAME or 10 μM NaN_3_ alone, or in the combination treatments as indicated for 6 h. Then, *CycA *(**a**), *CycB *(**b**), *CDKA *(**c**) and *CDKB *(**d**) expression levels were analyzed by real-time RT-PCR. The expression levels of the genes were presented as values relative to water treatment (Con). Values (means ± SE) are the averages of three independent experiments. Bars with different letters were significantly different in comparison with the control at *P* < 0.05 according to Duncan’s multiple test

To examine the molecular mechanism of HRW-induced phase changes during adventitious root development, the changes in the expression of two cyclins (*CycA and CycB*) and two CDK (*CDKA* and *CDKB*) genes were analyzed by qRT-PCR. As shown in Fig. 5a, HRW and SNP increased the expression of *CycA* by 15.14 and 26.28 % in comparison with the control, respectively. However, compared with HRW treatment, the expression of *CycA* was significantly reduced by 83.00, 67.70 and 98.90 % in the treatments of HRW + cPTIO, HRW + L-NAME and HRW + NaN_3_ (Fig. 5a). Figure 5b showed that HRW and SNP treatments increased the expression of *CycB* to 138.83 and 148.11 % of the control. Subsequent observation showed that the applications of cPTIO, L-NAME and NaN_3_ were able to down-regulate HRW-mediated expression of *CycB* by 64.97, 54.50 and 96.30 %, respectively (Fig. 5b). The transcript level of *CDKA* in explants treated with HRW and SNP increased and reached a maximum 145.38 and 164.21 % increase compared to the control. However, when, cPTIO, L-NAME and NaN_3_ were administered to HRW-treated explants, it resulted 26.96, 20.44, and 23.51 % reduction in the expression of *CDKA* (Fig. 5c). Meanwhile, the higher transcript levels of *CDKB* was observed in the HRW and SNP treatments, which were 143.72 and 163.95 % higher than that of controls. In addition, cPTIO, L-NAME and NaN_3_ decreased the transcription levels of the *CDKB* in HRW treatment (Fig. 5d). These findings suggested that NO might be involved in H_2_-induced the expression of cell cycle regulatory genes.

### NO was responsible for HRW-induced the expression profiles of CsDNAJ-1,CsDPK1 and CsCDPK5 during adventitious root development

**Fig. 6:**
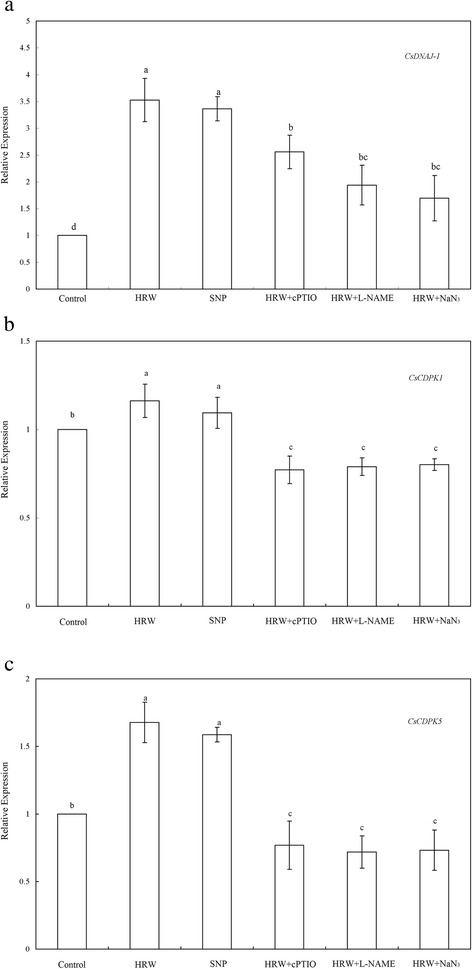
Effect of HRW, SNP, cPTIO, L-NAME and NaN_3_ on the expression profiles of *CsDNAJ-1*(**a**), *CsDPK1*(**b**), *CsCDPK5*(**c**) in the induction of adventitious rooting. The primary root system was removed from hypocotyls of 5-day-old germinated cucumber. Explants of cucumber were incubated with 50 % HRW, 50 μM SNP, 200 μM cPTIO, 30 μM L-NAME or 10 μM NaN_3_ alone, or in the combination treatments as indicated for 24 h. Then, relative gene expression was analyzed by real-time RT-PCR. The expression levels of the genes were presented as values relative to water treatment (Con). Values (means ± SE) are the averages of three independent experiments. Bars with different letters were significantly different in comparison with the control at *P* < 0.05 according to Duncan’s multiple test

Furthermore, *CsDNAJ-1*, *CsCDPK1* and *CsCDPK5* gene expression was used as a molecular probe to investigate the molecular mechanism of HRW-induced adventitious rooting. Compared with the control, HRW and SNP treatment were able to induce higher expression of the *CsDNAJ-1*, *CsCDPK1* and *CsCDPK5* during the first 24 h period of treatments (Fig. 6). These expressions were well matched with the number and length of adventitious root observed after another 4 days of treatment. It was interesting to note that the expression of *CsDNAJ-1* induced by HRW and SNP was 252.67 and 236.35 % higher than the control treatment (Fig. 6a). The *CsCDPK1* and *CsCDPK5* transcript level was induced by HRW and SNP also higher than that the control (Fig. 6 a and b). The co-treatment of HRW and cPTIO down-regulated the transcript levels of *CsDNAJ-1*, *CsCDPK1* and *CsCDPK5* by 27.46, 33.56 and 54.20 %, respectively (Fig. 6). Meanwhile, if L-NAME was administered to HRW-treated explants, it also resulted in a reduction in these genes expression. Compared with HRW treatment, HRW + NaN_3_ treatment significantly decreased the transcript levels of these genes (Fig. 6). Thus, the HRW-induced expression of *CsDNAJ-1*, *CsCDPK1* and *CsCDPK5* genes was significantly inhibited by cPTIO, L-NAME and NaN_3_. The above results further strengthened the hypothesis that NO might be, at least partially, involved in HRW-induced adventitious root development.

## Discussion

Adventitious roots play important roles in nutrient and water uptake in plant; and their formation is widely used for plant clonal propagation. Previous studies have shown the role and relationship between H_2_ and NO in plant stress response [32] and stomatal closure [37]. However, there is little research in the crosstalk between H_2_ and NO during adventitious rooting. Here, we focus on the involvement of NO H_2_-induced in cell cycle activation during adventitious rooting.

H_2_ has aroused worldwide attention because of its selective reduction [25]. Accumulating evidence indicates that H_2_ is a new signaling molecular and plays important roles in plants. In the present study, we illustrated that HRW, when applied exogenously, enhanced the number and length of adventitious root in a dose-dependent manner (Fig. 1). These findings are also consistent with a previous report showing that exogenous H_2_ was able to regulate cucumber adventitious root development [12]. Much research has focused on the physiological roles of H_2_ in plant responses against salt stress [27, 28], cadmium toxicity [31], mercury toxicity [33], paraquat-induced oxidative stress [30] and aluminum stress [32]. Ample evidences have shown that NO classified as a gasotransmitter was able to induce adventitious rooting [23]. We have shown previously that NO, when applied exogenously, also promoted the formation of adventitious root in marigold [10]. NO was found to promote adventitious root development in *Panax ginseng* by the generation of O_2−_ [42]. In order to ascertain whether NO signaling pathway is involved in H_2_-induced adventitious root, NO scavenger cPTIO and inhibitors L-NAME tungstate and NaN_3_ were used in the experiment. We found that cPTIO, L-NAME tungstate and NaN_3_ caused partial inhibition of HRW-induced adventitious root development (Fig. 2). In view of the inhibitory roles of NO-scavenger, NOS-inhibitor and NR-inhibitor in HRW-induced response, NO might be an essential gas signaling molecule in H_2_-mediated adventitious rooting. Until now, the relationship between H_2_ and NO both in animals and plants still remains to be elucidated. Although H_2_ could not inhibit intracellular NO production, it significantly suppressed NO-induced cytotoxicity in PC12 cells [43]. In alfalfa, H_2_ alleviated aluminum stress via decreasing NO production [32]. Xie et al. [37] demonstrated that NO production was contributed to H_2_-promoted stomatal closure in Arabidopsis. Our results also showed that when NO production was blocked, the promotive roles of HRW in adventitious rooting were reversed (Fig. 2). These results indicate that NO may act as a downstream signaling molecule involved in H_2_-induced adventitious root development. However, further studies are needed to determine the crosstalk between H_2_ and NO in different physiological processes.

Previous animal researches showed that there is a relationship between H_2_ and NO in some situations [36, 43]. In plants, Xie et al. [37] discovered that exposure of Arabidopsis to HRW resulted in an increase in NO content. Additionally, the effect of H_2_ in alleviating aluminum-induced inhibition of root elongation in alfalfa may derive from the decrease of NO [32]. In addition, an increase in H_2_-mediated NO production catalyzed by NOS-like protein and NR might be required and be part of the molecular events involved in H_2_ action. Here, our evidence supports the possibility that NOS-like- and NR-dependent NO production contributes to H_2_-promoted adventitious root development in cucumber (Fig. 3). Coincidently, it has been demonstrated that NO levels are apparent during the H_2_-induced stomatal closure [37]. It has also been found opposite observation that H_2_ decreased NO production in aluminum-induced inhibition of root elongation [32]. Therefore, present researches indicate that there is a complex interaction between H_2_ and NO in some physiological processes.

Previous studies have shown that cell cycle regulation in the xylem pericycle may play crucial roles in root organogenesis [44, 45]. Cell cycle regulation occurred in the xylem pericycle, in which cells proceed to G2 phase, whereas the rest of pericycle remained at G1 phase [46]. Here, our analyses indicated that that H_2_- and NO-induced an accumulation of cells in the S phase during adventitious rooting, indicating that H_2_- and NO-induced cell cycle activation contributed to rooting (Fig. 4). It has been previously reported that cell cycle induction played a key function in adventitious root growth [45] and lateral root formation [47]. This is the first report to show that H_2_ and NO are involved in cell cycle progression during adventitious root development. Interestingly, cPTIO, L-NAME, and NaN_3_ all partially reduced H_2_-induced accumulation of cells in S phase and then inhibited the adventitious root development (Fig. 4). It is generally accepted that plant hormones play a central role in the reactivation of the cell cycle during root development [48]. The inducible effect of NO on cell cycle activation has been reported in tomato lateral formation [47]. The authors noted that auxin-dependent cell cycle gene regulation might be dependent on NO. Thus, our pharmacological evidence supports the possibility that, at least in our experimental condition, H_2_ and NO might form a linear signaling pathway in regulating cell cycle activation during adventitious rooting.

Cell-cycle regulatory genes have been shown to be involved in regulating cell division in internode growth [49] and root meristem induction [45]. To understand the mechanisms of NO involved in H_2_-regulated cell cycle that resulting in root initiation, detailed molecular studies were conducted in this study. The genes involved in the transition of G1 to S, *CycA*, *CycB*, *CDKA* and *CDKB* were significantly up-regulated by NO and H_2_ treatments (Fig. 5), which consistent with the phase change of cell cycle. Otvos et al. [50] reported that NO promoted cell division and embryogenic cell formation in leaf protoplast-derived cells of alfalfa. NO transiently induced *CyCA2;1* and *CYCD3;1* mRNA expression in cell suspensions. These results correspond well with the expression of *CycA* and *CycB* in our experimental condition. Previous study also found that NO mediated the induction of the *CYCD3;1* gene at the beginning of lateral root primordial formation [47]. Thus, these results validate the involvement of NO in regulating cell cycle-related genes during lateral and adventitious rooting. It is noteworthy that the expression of *CDKA* was markedly up-regulated by H_2_ and NO, suggesting that A-type CDKs may play a main role in G1-to-S transition. Himanen et al. [44] also found that the transcription level of *CDKA* was induced in auxin-mediated cell cycle activation during early lateral root initiation. The level of the *CDKA* transcript remained at high levels in NO treatment during lateral rooting indicating that pericycle cells may be competent for cell division in tomato [47]. Additionally, our data showed that the transcript level of *CDKB* was less than that of *CDKA* after H_2_ treatment. It may because B-type CDKs regulates cell cycle progression to the mitotic phase, G2 to M [51]. This study further showed that the transcript levels of *CycA*, *CycB*, *CDKA* and *CDKB* induced by H_2_ were partially inhibited by the NO scavenger cPTIO, NOS inhibitor L-NAME and NR inhibitor NaN_3_ (Fig. 5). Thus, the transcript profile presented here allows a simple model to be proposed that NO may be involved in cell cycle activation during H_2_-induced adventitious rooting. The evidence provided here further confirms that H_2_ and NO may specifically regulate cell cycle activation and adventitious root formation. Furthermore, NO may be downstream signal molecule during H_2_ –induced adventitious rooting, present in competent pericycle cells, which finally activates cell cycle-related genes.

It has been observed that the target genes of adventitious root development, *CsDNAJ-1*, *CsCDPK1* and *CsCDPK5* could be induced by auxin, CO [8], and hydrogen sulfide [11]. All DnaJ-like proteins characterized by a J domain regulated interactions with Hsp70 in protein folding and the assembly and disassembly of protein complexes. It has illustrated that the genes of DnaJ-like protein (s) and calcium-dependent protein kinases (CDPKs) were involved in the initiation and development of adventitious root [8, 52]. Additionally, IAA and NO induced the activity of CDPK that associated with cell differentiation, division, and /or differentiation during the formation of adventitious root [5]. In this study, the molecular evidence showed that H_2_ and NO treatments both induced high expression of the *CsDNAJ-1*, *CsCDPK1* and *CsCDPK5* genes (Fig. 6), which were consistent with the number of adventitious roots observed. Bai et al. [52] found that IAA, 3-O-C10-HL and H_2_O_2_ increased the expression of *CDC2*, *ARC2*, and *CDPK* as well as the Aux/IAA gene family members *AUX22c*, *AUX22d*, and AUX22e in mung bean. Recently, Lin et al. [12] also reported that H_2_ induced the expression of target genes related to adventitious root formation in a heme oxygenase-1/carbon monoxide-dependent manner during cucumber adventitious rooting. Application of NO scavenger cPTIO, NOS inhibitor L-NAME and NR inhibitor NaN_3_ were able to down-regulate H_2_-induced transcription levels of *CsDNAJ-1*, *CsCDPK1* and *CsCDPK5* (Fig. 6). Thus, we deduced that H_2_-induced *CsDNAJ-1*, *CsCDPK1* and *CsCDPK5* expression might be regulated by NO production. These findings might suggest that *CsDNAJ-1*, *CsCDPK1* and *CsCDPK5* expression at the earlier state might be required for H_2_-induced adventitious root development in a NO-independent manner.

## Conclusion

**Fig. 7:**
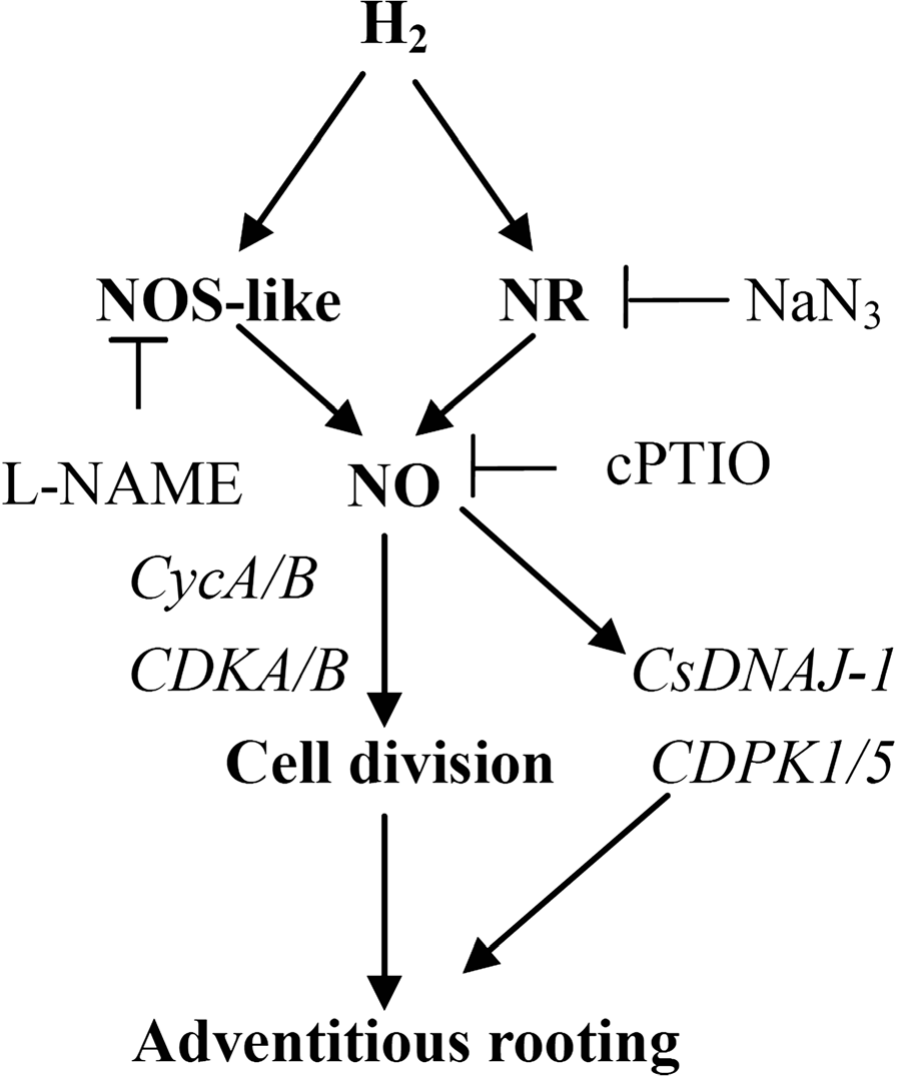
Schematic model of the signaling networks involving H_2_ and NO during adventitious root development in cucumber. The H_2_ triggers a transient NO accumulation. In turn, NO induces the division of cell and the expression of cell cycle-related gens, *CycA/B* and *CDKA/B*. The above pathway might be mediated by the expression of *CsDNAJ-1* and *CDPK1/5* genes. T bars, inhibition

Taken together, our results suggested that both H_2_was involved in adventitious root development and NO might be downstream signal molecules in the H_2_ signalling cascade. Meanwhile, the evidence presented here indicated that H_2_ activated cell cycle and up-regulated cell cycle-related genes and target genes related to adventitious rooting via NO pathway (Fig. 7). However, the network responsible for adventitious root development induced by H_2_ and NO may be very complex. Therefore, considerably more work will be done for further investigation of the molecular mechanism underlying H_2_- and NO-induced adventitious rooting.

## Methods

### Plant material and growth conditions

Cucumber (*Cucumis sativus* ‘Xinchun 4’) seeds were kindly supplied by Gansu Academy of Agricultural Sciences, Lanzhou, China. Selected identical seeds were germinated in Petri dishes on filter papers soaked in distilled water, then transferred to an illuminating incubator and maintained at 25 ± 1 °C for 5d with a 14-h photoperiod (photosynthetically active radiation = 200 μmol m^−2^ s^−1^). Primary roots of 5-d-old seedlings were removed and the cucumber explants were then maintained under the same temperature and photoperiod conditions described above for another 5 d in the presence of different media as indicated below. Then, records were taken of root number per explants, root length. Meanwhile, corresponding photographs were taken.

### Treatments of explants, chemicals

After primary roots were removed, every ten cucumber explants were put into a Petri dish containing 6 ml of distilled water, different concentration of hydrogen-rich water (HRW) as indicated in Fig. 1. 30 μM N-nitro-L-arginine methyl ester (L-NAME, Sigma, USA) or 200 μM 2-(4-carboxyphenyl)-4,4,5,5-tetramethylimidazoline-1-oxyl-3-oxide (cPTIO, Sigma, USA), or 100 μM tungstate or 10 μM NaN_3_ were added together with optimum concentration of HRW. 200 μM cPTIO was added together with optimum concentration of SNP. Inactivated SNP (50 μM) had previously been exposed to light to drive off NO about 24 h. The concentration of these chemicals was selected based on the results of preliminary experiment and previous experiment [10]. The solutions were prepared in complete darkness, and immediately diluted to the demanded concentrations at pH values of 6.5. Unless stated otherwise, the remaining chemicals were of analytical grade from Chinese companies.

### Preparation of hydrogen-rich water (HRW)

Purified H_2_ gas (99.99 %, *v/v*) generated from a hydrogen gas generator (QL-300, Saikesaisi Hydrogen Energy Co., Ltd., China) was bubbled into 2 l distilled water at a rate of 300 ml min^−1^ for 30 min. Then, the corresponding HRW was rapidly diluted to required saturations (1, 10, 50 %, [*v/v*]). In our experimental conditions, H_2_ concentration in freshly prepared HRW determined with a “Dissolved hydrogen portable meter” (Trustlex Co., Led, ENH-1000, Japan) was 0.45 mM, and maintained at a relative constant level in 25 °C for at least 12 h.

### Determination endogenous NO content

NO content was determined using the Greiss reagent method [3] with some modifications. Samples of cucumber hypocotyl (0.2 g) were frozen in liquid nitrogen, then ground in a mortar and pestle in 4 mL of 50 mM ice-cold acetic acid buffer, pH 3.6, containing 4 % (*w/v*) zinc diacetate. The homogenates were centrifuged at 10,000 × *g* for 15 min at 4 °C, and the supernatants were collected. For each sample, 0.1 g charcoal (Shanghai Chemical Reagent Co. Ltd.) was added. After vortex mixing and filtration, the filtrate was leached and collected. A mixture of 1 mL of filtrate and 1 mL of Greiss reagent was incubated at room temperature for 30 min to concert nitrite into a purple azo-dye. The absorbance was then assayed at 540 nm. NO content was calculated by comparison to a standard curve of NaNO_2_.

### Hypocotyl cell nucleus extracts

Cucumber hypocotyls were excised about 0.5 cm and fixed in 4 % formaldehyde (0.1 M phosphate buffer, pH 7.4) for 15 min. Then the hypocotyls were cut up with blade in Galbraith butter (45 mM MgCl_2_, 30 mM sodium citrate, 20 mM 4-morpholinopropanesulfonic acid). Finally, cell nucleus suspension was collected with 30 μm mesh screen in 5 ml tube.

### Fluorescence microscopic image analysis of the cell cycle

Microscopy was conducted as described by [53] with modifications. Cell nucleus suspension added fluorochrome DAPI (1:100000, Molecular probe), 0.1 % Triton-X 100 and 50 μg mL^−1^ Rnase1 (DNase-free, Qiagen) was observed and taken photos with fluorescence microscope (Leica 400×, Planapo, Wetzlar, Germany). Microscopic photography conditions setting value was fixed and no excessive exposure of the nucleus. Each sample was used to analysis at least 1000 the fluorescence microscopic images of nucleus. The fluorescence intensity of nucleus (grey value) was measured with the help of Image Pro software (Media Cyberntics, USA).

### RNA extraction

Total RNA was extracted from about 200 mg (fresh-weight) excised cucumber hypocotyl (5 mm) 6 h and 24 h after treatment using TRIZOL reagent (Sangon, China) according to the manufacturer’s instructions.

### Transcript level estimation with qRT-PCR

Quantitative Real-time PCR (qRT-PCR) reactions were performed using an ABI StepOne Plus system (Applied Biosystems, Carlsbad, CA) along with Qiagen Quantifast SYBR Green PCR Kit (Huaxia Ocean Science and Technology Con., Ltd., China). Gene-specific primers of cell cycle-related genes (at 6 h after treatment) and target genes (at 24 h after treatment) responsible for adventitious rooting for qRT-PCR were amplified using the primers in Table 1. Each reaction (20 μl total volume) consisted of 10 μl iQ SYBR Gree Supermix, 1 μl of diluted cDNA and 0.4 μl of forward and reserve primers. PCR cycling conditions were as follows: 5 min at 95 °C followed by 40 cycles of 10 s at 95 °C and 30 s at 60 °C with data collection at the annealing step. After the 40 cycles, we included a dissociation/melting curve stage with 15 s at 95 °C, 60 s at 60 °C, and 15 s at 95 °C. The cucumber *actin* gene was used as an internal control. The calculation of relative gene expression was conducted as described by Livak and Schmittgen [54].

**Table 1:** Primers used for qPT-PCR assays

Gene	Accession no.	Primer pairs
*CycA*	EW968279	F: 5′- GCCTCTGCTGTAACAACACTCAT-3′
		R: 5′- TGTGCTGGCTGTATTTTTCTCTG -3′
*CycB*	EW968280	F: 5′- AATGAGGGCTATTTTGGTGGA-3′
		R: 5′- TATCCGAAAGGCACACAAAGTC-3′
*CDKA*	EW968281	F: 5′- ATCTAAAACCCCAAAATCTGCT-3′
		R: 5′- CAAATGCTCTTGCCAGTCC -3′
*CDKB*	EW968282	F: 5′-CAATCCCTCTATGTCGTTCG-3′
		R: 5′- CAAATGCTCTTGCCAGTCC-3′
*CsDNAJ-1*	X67695	F: 5′- GACCACTCTCCACGATGTCAAC-3′
		R: 5′- ATCAATGTGTTATGGCGGTAGC-3′
*CDPK1*	AJ312239	F: 5′- GGAGTTGGAAGGAGGACGATG-3′
		R: 5′- TGAGATTTAGCAGTAAGGACGC-3′
*CDPK5*	AY02785	F: 5′- ATGAGGAAAGGCAATCAGGAAT-3′
		R: 5′- AAAGAAGCACATAAAATCAAGCAGA
*actin*	DQ641117	F: 5′-CCCATCTATGAGGGTTACGCC-3′
		R: 5′-TGAGAGCATCAGTAAGGTCACGA-3′

### Statistical analysis

Where indicated, results were expressed as the mean values ± SE of at least three independent experiments. Statistical analysis was performed using the Statistical Package for Social Sciences for Windows (version 13.00; SPSS, Inc., Chicago, IC, USA). For statistical analysis, Duncan’s multiple test (*P* < 0.05) was chose as appropriate.

## Abbreviations

CDKA, cyclin-dependent kinase A; CDKB, cyclin-dependent kinase B; CycA, A-type cyclin; CycB, B-type cyclin; DAPI, 4′, 6-diamidino-2-phenylindole; H_2_, hydrogen gas; H_2_O_2_, hydrogen peroxide; HRW, hydrogen-rich water; LI SNP, Light-inactivated SNP; L-NAME, NO synthase (NOS)-like enzyme inhibitor N^G^ –nitro-_L_-arginine methylester hydrochloride; NO, nitric oxide; SNP, souium nitroprusside; cPTIO, 2-(4-carboxyphenyl)-4,4,5,5-tetramethylimidazoline-1-oxyl-3-oxide potassium salt
